# Serum Serine and the Risk of All-Cause Mortality: A Nested Case-Control Study From the China Stroke Primary Prevention Trial (CSPPT)

**DOI:** 10.3389/fnut.2022.946277

**Published:** 2022-07-12

**Authors:** Qiangqiang He, Nan Zhang, Qiongyue Liang, Zhuo Wang, Ping Chen, Yun Song, Ziyi Zhou, Yaping Wei, Yong Duan, Binyan Wang, Peiwu Qin, Xianhui Qin, Xiping Xu

**Affiliations:** ^1^Graduate School at Shenzhen, Tsinghua University, Shenzhen, China; ^2^Shenzhen Evergreen Medical Institute, Shenzhen, China; ^3^Department of Cardiology, Peking University First Hospital, Beijing, China; ^4^State Key Laboratory of Natural Medicines, Research Center of Biostatistics and Computational Pharmacy, China Pharmaceutical University, Nanjing, China; ^5^Key Laboratory of Precision Nutrition and Food Quality, Ministry of Education, Department of Nutrition and Health, College of Food Sciences and Nutritional Engineering, China Agricultural University, Beijing, China; ^6^College of Pharmacy, Jinan University, Guangzhou, China; ^7^AUSA Research Institute, Shenzhen AUSA Pharmed Co. Ltd., Shenzhen, China; ^8^Institute of Biomedicine, Anhui Medical University, Hefei, China; ^9^Yunnan Key Laboratory of Laboratory Medicine, Kunming, China; ^10^Department of Clinical Laboratory, the First Affiliated Hospital of Kunming Medical University, Kunming, China; ^11^National Clinical Research Center for Kidney Disease, State Key Laboratory for Organ Failure Research, Guangdong Provincial Key Laboratory of Renal Failure Research, Guangzhou Regenerative Medicine and Health, Guangdong Laboratory, Division of Nephrology, Nanfang Hospital, Southern Medical University, Guangzhou, China; ^12^Institute of Biopharmaceutical and Health Engineering, Shenzhen International Graduate School, Tsinghua University, Shenzhen, China

**Keywords:** serum serine, longitudinal cohort, all-cause mortality, hypertension, nutrition

## Abstract

**Background:**

Serine plays a key role in numerous cellular processes, the levels and metabolism is therefore of critical importance. However, few data are available to illustrate the association of serine with long-term health effects, especially, the predictive value for long-term mortality.

**Objective:**

This study was conducted to evaluate the relationship between serum serine levels and all-cause mortality in general hypertensive patients in a longitudinal cohort, and to examine the potential effect modifiers.

**Methods:**

A nested case-control (NCC) study was conducted utilizing 20702 hypertensive participants from the China Stroke Primary Prevention Trial (CSPPT), a randomized, double-blind, actively controlled trial conducted from May 2008 to August 2013 in China. The current study included 291 cases of all-cause mortality and 291 controls matched on age (≤ 1 year), sex and treatment group. All-cause mortality was the main outcome in this analysis, which included death due to any reason.

**Results:**

With the increase in serum serine levels, the risk of all-cause mortality first increased before flattening. After adjusting for related variables, the risk of mortality increased significantly with the increase of serum serine levels. Compared with group Q1, the mortality risk of group Q2, Q3 and Q4 were significantly increased [ORs, 95% CI: Q2: 2.32, (1.32–4.07); Q3: 2.59, (1.48–4.54); and Q4: 1.85, (1.07–3.22)]. In the exploratory analysis, we observed three effect modifiers, total homocysteine, 5-Methyltetrahydrofolate, and estimated glomerular filtration rate significantly modified the serum serine and all-cause mortality association.

**Conclusion:**

Serum serine levels were significantly associated with an increased risk of all-cause mortality in hypertensive patients. Our results and findings, if confirmed further, suggest that serum serine should be considered as a marker for screening risk factors of mortality.

**Clinical Trial Registration:**

[https://www.clinicaltrials.gov/ct2/show/study/NCT00794885.], identifier [CSPPT, NCT00794885].

## Introduction

In addition to contributing to protein synthesis, amino acids support various bioenergetic and biosynthetic processes in mammalian cells. Serine, the main source of one-carbon donors (1), plays a key role in feeding one-carbon units to the tetrahydrofolate (THF) cycle and supports both nucleotide synthesis (2) and contributes to the S-adenosyl methionine (SAM) cycle (3) by providing formyl groups, thus, the dysregulation of serine metabolism has an impact on DNA methylation (4) and epigenetics (5).

Serine deficiency disorders are usually caused by defects in the synthesizing enzymes of the serine biosynthesis pathway, the biochemical hallmarks of synthesizing enzymes defects are low concentrations of serine in cerebrospinal fluid and plasma (6). However, aberrant elevated serine levels were observed in the type 1 diabetes subjects (7) and associated with decreased overall survival (OS) in head and neck cancer (HNC) patients (8). Besides, there are evidence that cancer cells usually demonstrate increased serine biosynthesis and uptake.

Different intake doses of some amino acids may be associated with the changes of the mortality (9), it was reported indispensable amino acids have a positive and some non-indispensable amino acids have a negative, independent, strong association with the risk of cardiovascular mortality (10). The prior study also demonstrated that plasma amino acid constellations are promising additional biomarkers for predicting mortality in end-stage liver disease (11). Numerous studies have focused on the mechanism of serine metabolism and its physiological function, however, few data are available to illustrate the association of serine with long-term health effects, especially, the predictive value for long-term mortality. We therefore, conducted a retrospective cohort nested case-control (NCC) study to investigate the associations of serum serine levels and the risk of all-cause death in a cohort of hypertensive adults.

## Methods

### Study Population

The methods and major results of the CSPPT (NCT00794885) have been reported elsewhere (12). Briefly, the CSPPT was a multi-community, randomized, double-blind, controlled trial conducted from 19 May 2008 to 24 August 2013 in 32 communities in Anqing and Lianyungang of China. Eligible participants were men and women aged 45–75 years with hypertension, defined as seated resting systolic blood pressure (SBP) ≥ 140 mm Hg or diastolic blood pressure (DBP) ≥90 mm Hg at both the screening and recruitment visits or who were taking antihypertensive medications. The major exclusion criteria included a history of physician-diagnosed stroke, myocardial infarction, heart failure, postcoronary revascularization, or congenital heart disease.

### Intervention and Follow-Up

In the CSPPT, a total of 20,702 eligible participants were randomly assigned, in a 1:1 ratio, to one of two treatment groups: a daily oral dose of one tablet containing 10 mg enalapril and 0.8 mg folic acid (the enalapril folic acid group), or a daily oral dose of one tablet containing 10 mg enalapril only (the enalapril-only group). Participants were followed up every 3 months for a median duration of 4.5 years. A total of 291 mortality cases from the CSPPT were analyzed in this study.

### Outcomes Assessment

All-cause mortality, a prespecified endpoint of the CSPPT, was the primary outcome of this analysis. All-cause mortality included mortality due to any reason. Evidence for mortality included death certificates from hospitals or reports to the investigator from follow-up visits. Secondary outcomes included death from cardiovascular disease (CVD) including sudden cardiac death, death due to MI, heart failure, stroke, or cardiovascular invasive procedures, death due to cardiovascular hemorrhage, death due to other known vascular causes, and death from cancer including death as a direct result of cancer, or from a complication of cancer, or withdrawal of other therapies due to concerns relating to the poor prognosis associated with cancer. All the study outcomes were reviewed and adjudicated by an independent Endpoint Adjudication Committee, whose members were unaware of study-group assignments.

### Nested Case-Control Study

During a median treatment duration of 4.5 years, all-cause mortality occurred in 302 participants (2.9%) in the enalapril-folic acid group as compared to 320 participants (3.1%) in the enalapril group (HR, 0.94; 95% CI, 0.81–1.10; *P* = 0.47).

**Figure 1:**
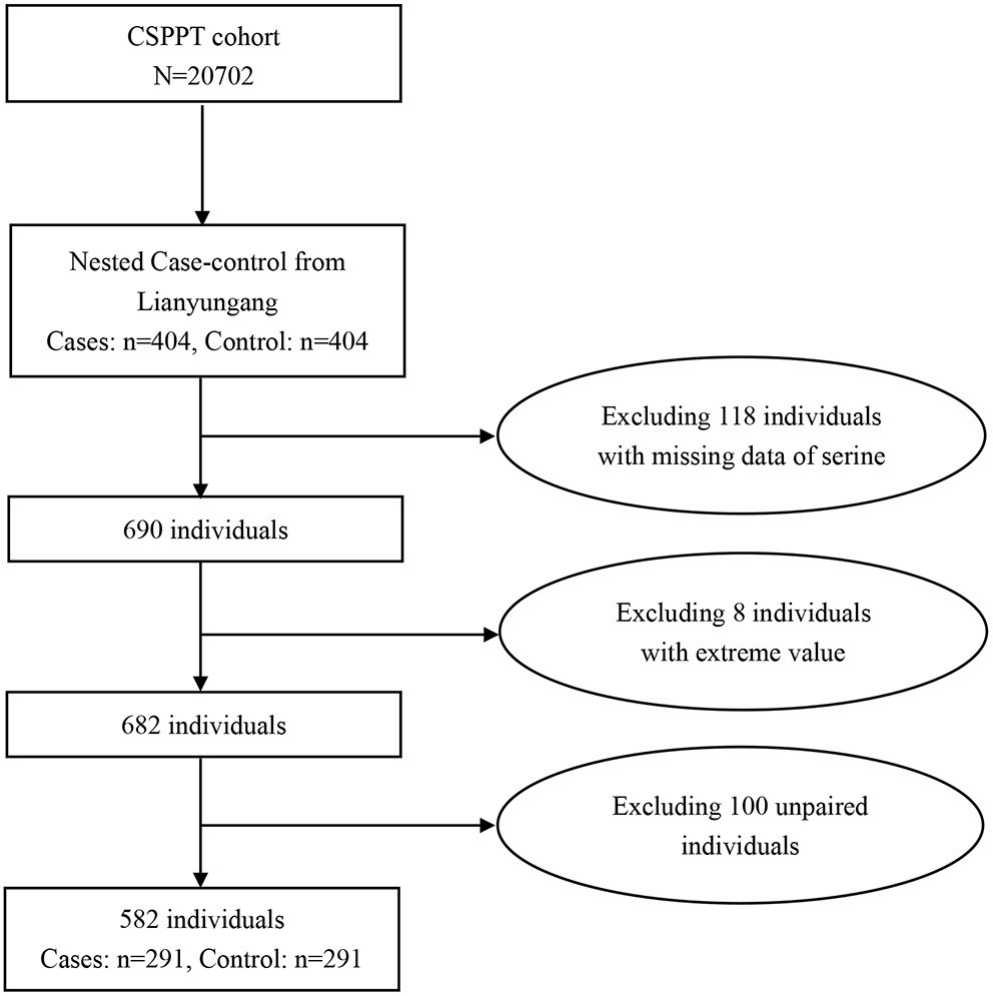
Flow chart of the study participants.

Using data from the CSPPT, we established a nested case-control study with a total of 291 incident cases and 291 matched controls within this cohort. Controls were randomly chosen from the baseline CSPPT participants who were alive during the follow-up and were matched for age (≤ 1 year), sex, treatment group, and study site with the cases on a 1:1 ratio. Our final analysis included 291 incident cases and matched them with 291 controls within this cohort from the study center Lianyungang, exclusions included 118 participants with missing data on serine, eight participants with extreme values of serine, and 100 unpaired participants (Figure 1).

The parent study (the CSPPT) and the current study were approved by the Ethics Committee of the Institute of Biomedicine, Anhui Medical University, Hefei, China (Federal wide assurance number: FWA00001263). All participants provided written informed consent.

### Laboratory Assays

Overnight fasting venous blood samples were obtained from each study participant at baseline. Routine biochemical samples, including serum total homocysteine (tHcy), blood glucose, and lipid levels, were analyzed on an automatic clinical analyzer (Beckman Coulter) at the core laboratory of the National Clinical Research Center for Kidney Disease, Guangzhou, China. Serum folate and vitamin B12 were measured in a commercial laboratory using a chemiluminescent immunoassay (New Industrial, Shenzhen). The estimated glomerular filtration rate (eGFR) was calculated with the use of the Chronic Kidney Disease Epidemiology Collaboration equation (13). The stable-isotope-dilution liquid chromatography-tandem mass spectrometry (4500MD, AB SCIEX) was used to detect serine concentrations in the electrospray ionization (ESI +) mode. The mobile phase was 0.5% acetic acid-water (containing 10 m mol/L ammonium acetate)−95% acetonitrile-water (containing 0.5% acetic acid, 10 m mol/L ammonium acetate). The chromatographic column used was Waters ACQUITY UPLC ^®^ BEH HILIC (2.1 × 100 mm, 1.7 μm). This method had excellent sensitivity (LOQ 1 ug / ml), precision (CV < 8%) and recovery (87–111%).

### Statistical Analysis

Baseline characteristics were presented as means ± SDs or median (interquartile range, IQR) for continuous variables and proportions for categorical variables. Differences in baseline characteristics between cases and controls were compared using the Chi-square test for categorical variables and the Wilcoxon signed rank test for continuous variables.

Odds ratios (ORs) and 95% confidence intervals (95% CIs) for all-cause mortality in relation to serum serine levels were calculated using conditional logistic regression models, without and with adjustment for age, sex, body mass index (BMI), smoking status, alcohol drinking status, SBP, DBP, fasting blood glucose, total cholesterol (TC), triglycerides (TG), methylenetetrahydrofolate reductase (MTHFR) C677T genotype, treatment group, high-density lipoprotein cholesterol (HDL-C), eGFR, serum folate, total homocysteine (tHcy), vitamin B12 at baseline, as well as time-averaged SBP and time-averaged DBP during the treatment period.

As additional exploratory analyses, possible modifications of the relation of all-cause mortality with serum serine were also assessed for variables including sex, age (<64.5 [median] vs. ≥64.5 years), treatment group (enalapril vs. enalapril-folic acid), MTHFR C677T genotype (CC vs. CT vs. TT), tHcy (<13.7 [median] vs. ≥13.7 μ mol/L), serum folate (<6.5 [median] vs. ≥6.5 ng/mL), 5-Methyltetrahydrofolate (5-MTHF, <6.1 [median] vs. ≥6.1 ng/mL), and vitamin B12 (<374.7 [median] vs. ≥374.7 p g/mL).

A 2-tailed *P* < 0.05 was considered to be statistically significant in all analyses. R software (version 3.6.1; http://www.R-project.org) and Empower (R) (www.empowerstats.com, X&Y Solutions, Inc. Boston, MA) were used for all statistical analyses.

## Results

### Baseline Characteristics

In this study, we analyzed 291 mortality cases, and 291 matched controls. Table 1 describes the baseline characteristics of this population. There were no significant differences in age, sex, BMI, treatment group, or smoking and drinking status between the cases and the controls. The mean time-averaged systolic blood pressure and diastolic blood pressure of the cases (144.7/83.4 mmHg) were significantly higher than those of the controls (140.4/82.2 mmHg). In addition, no significant differences were found in laboratory parameters including fasting blood glucose, blood lipids, baseline Hcy levels, baseline serum folate levels, baseline vitamin B12 levels, and eGFR between the two groups, nor was there any difference in MTHFR C677T genotypes between the two groups. But cases have significantly higher serum 5-MTHF concentrations compared to controls (*P* = 0.008). The median values of serum serine concentrations in mortality cases and control subjects were 325.5 μ mol/L (IQR: 275.1– 405.8) and 320.7 μ mol/L (IQR: 237.9–395.6), respectively, the levels for cases were significantly higher than those in the controls (*P* = 0.001).

**Table 1:** Baseline characteristics of the study participants stratified by control and case.

**Characteristics**	**Total (*N* = 582)**	**Mortality**		***P***
		**Controls (*n* = 291)**	**Cases (*n* = 291)**	
Age,y	64.5 (57.4, 70.6)	64.5 (57.4, 70.6)	64.5 (57.4, 70.7)	0.978
Male, *n* (%)	328 (56.4)	164 (56.4)	164 (56.4)	1.000
BMI, kg/m^2^	24.5 (22.2, 27.1)	24.9 (22.5, 27.3)	24.3 (21.9, 26.7)	0.060
**Treatment group**, ***n*** **(%)**				1.000
Enalapril	310 (53.3)	155 (53.3)	155 (53.3)	
Enalapril-folic acid	272 (46.7)	136 (46.7)	136 (46.7)	
**BP, mmHg**				
SBP at baseline	168.0 (156.0, 182.0)	165.3 (154.7, 180.7)	170.7 (158.0, 183.7)	0.073
DBP at baseline	94.0 (87.3, 101.3)	93.3 (87.7, 100.0)	95.3 (87.3, 102.0)	0.224
Time-averaged SBP	141.6 (134.7, 149.1)	140.4 (134.6, 146.9)	144.7 (135.0, 152.9)	0.001
Time-averaged DBP	82.6 (77.4, 88.3)	82.2 (77.5, 87.2)	83.4 (77.3, 89.8)	0.046
**Laboratory results**				
Fasting glucose, mmol/L	5.6 (5.2, 6.4)	5.6 (5.2, 6.3)	5.7 (5.2, 6.4)	0.450
Total cholesterol, mmol/L	5.6 (4.8, 6.3)	5.6 (4.9, 6.3)	5.5 (4.6, 6.2)	0.410
Triglycerides, mmol/L	1.4 (1.1, 2.0)	1.5 (1.0, 2.1)	1.4 (1.1, 1.9)	0.352
HDL cholesterol, mmol/L	1.3 (1.1, 1.5)	1.3 (1.1, 1.5)	1.3 (1.0, 1.6)	0.878
tHcy, μmol/L	13.7 (11.0, 18.8)	13.6 (10.8, 18.2)	14.0 (11.1, 19.3)	0.240
Folate, n g/mL	6.5 (4.8, 9.2)	6.8 (4.7, 9.3)	6.2 (4.9, 8.8)	0.848
5-MTHF, ng/mL	6.1 (3.4, 9.6)	6.7 (3.7, 10.3)	5.6 (3.1, 9.0)	0.008
Vitamin B12, pmol/L	374.7 (313.1, 460.2)	373.7 (312.7, 471.1)	374.8 (313.3, 448.9)	0.859
Serine, μmol/L	313.5 (254.2, 397.8)	302.7 (237.9, 395.6)	325.5 (275.1, 405.8)	0.001
eGFR, mL/(min per 1.73 m^2^)	91.2 (82.5, 99.2)	91.2 (82.4, 98.9)	91.3 (83.6, 99.7)	0.966
**MTHFR genotype**, ***n*** **(%)**				0.679
CC	141 (24.2)	73 (25.1)	68 (23.4)	
CT	292 (50.2)	148 (50.9)	144 (49.5)	
TT	149 (25.6)	70 (24.1)	79 (27.1)	
**Smoking**, ***n*** **(%)**				0.962
Never	316 (54.3)	158 (54.3)	158 (54.3)	
Former	70 (12.0)	36 (12.4)	34 (11.7)	
Current	196 (33.7)	97 (33.3)	99 (34.0)	
**Drinking**, ***n*** **(%)**				0.564
Never	332 (57.0)	163 (56.0)	169 (58.1)	
Former	65 (11.2)	30 (10.3)	35 (12.0)	
Current	185 (31.8)	98 (33.7)	87 (29.9)	

*Values are presented as median (Q1, Q3) or n (%). Differences between cases and controls in baseline characteristics were compared with the use of the Chi-square test for categorical variables and the Wilcoxon signed rank test for continuous variables. BP, blood pressure; SBP, systolic blood pressure; DBP, diastolic blood pressure; HDL, high-density lipoprotein; tHcy, total homocysteine; 5-MTHF, 5-methyl-tetrahydrofolate; eGFR, estimated glomerular filtration rate; MTHFR, methylenetetrahydrofolate reductase*.

### Association of Baseline Serine Levels With the Risk of All-Cause Mortality

**Figure 2:**
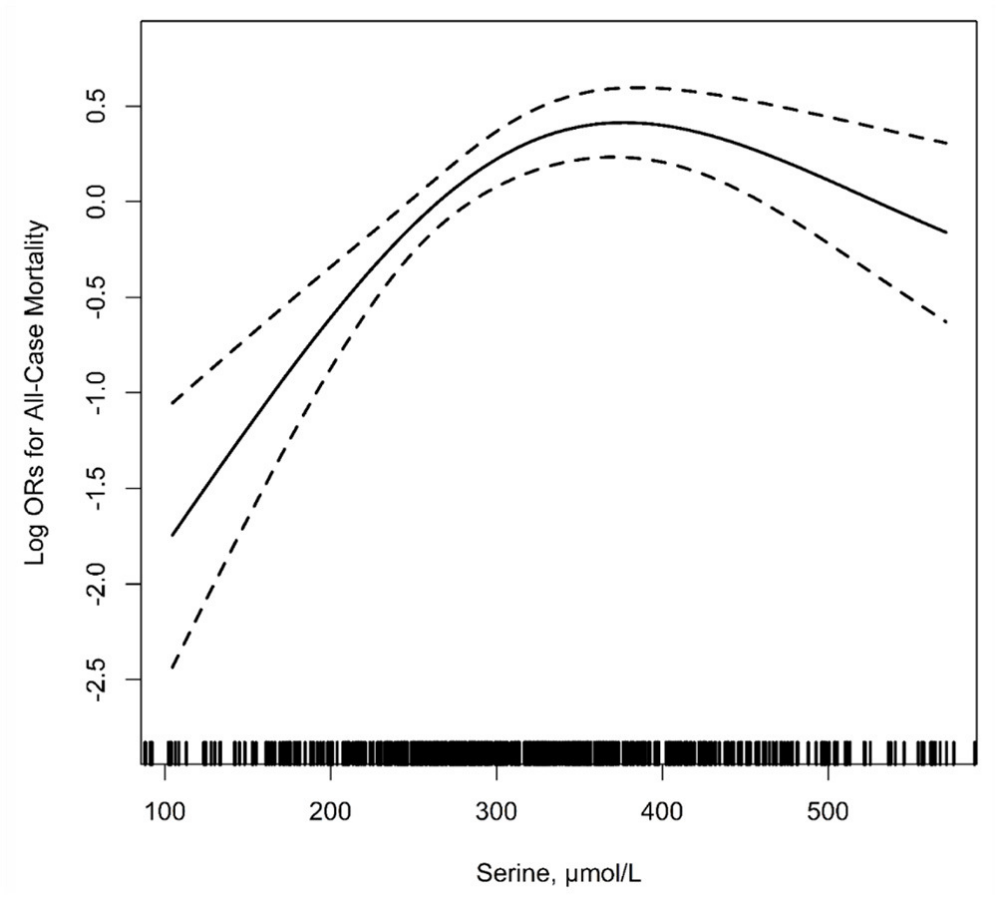
The relationship of serum serine with the risk of all-cause mortality^1^. ^1^Adjusted for age, sex, body mass index (BMI), treatment group, *MTHFR* C677T genotypes, smoking, alcohol drinking, systolic blood pressure (SBP), diastolic blood pressure (DBP), fasting blood glucose, total cholesterol (TC), triglycerides (TG), high-density lipoprotein cholesterol (HDL-C), estimated glomerular filtration rate (eGFR) at baseline, folate, total homocysteine (tHcy), vitamin B12 as well as mean SBP and DBP during the treatment period.

Figure 2 shows the relationship between serum serine and the risk of all-cause mortality. After adjusting for the related variables, as serine levels increased, the risk of mortality increased first and then tended to flatten. The results of logistic regression analyses are shown in Table 2, in the unadjusted model, with the increase of serum serine levels, the risk of all-cause mortality showed an overall upward trend. After adjusting for related variables, the risk of mortality increased significantly with the increase in serum serine levels. Serine was assessed as quartiles, compared with group the lowest quartile Q1, < 254.2 μ mol/L), the adjusted ORs (95% CI) for mortality of group Q2 (254.2–313.5 μ mol/L), Q3 (313.5–397.8 μ mol/L) and Q4 (≥ 397.8 μ mol/L) were significantly increased [ORs: Q2, 2.32 (1.32–4.07); Q3, 2.59 (1.48–4.54); Q4, 1.85 (1.07–3.22). *P* for trend =0.020]. When combined, the subjects in the up three quartiles [quartiles 2–4 (≥ 254.2 μ mol/L): the adjusted OR: 2.24; 95% CI: 1.52–3.30] showed a significantly higher risk of all-cause mortality compared with Q1 (*P* < 0.001).

**Table 2:** The risk of all-cause mortality with serum serine concentrations^1^.

	***N***	**Cases (%)**	**Crude model**		**Adjusted model^2^**	
			**OR(95%CI)**	***P*** **-value**	**OR(95%CI)**	***P*** **-value**
**Serine, μmol/L**	582	291(50.0)	1.24(1.03,1.50)	0.022	1.18(0.96,1.44)	0.112
**Quartile**						
Q1 (<254.2)	146	50 (34.2)	1.00 (1.00,1.00)	Ref	1.00 (1.00,1.00)	Ref
Q2 (254.2–313.5)	145	81 (55.9)	2.35 (1.44,3.83)	<0.001	2.32 (1.32,4.07)	0.004
Q3 (313.5–397.8)	145	83 (57.2)	2.43 (1.51,3.92)	<0.001	2.59 (1.48,4.54)	<0.001
Q4 (≥397.8)	146	77 (52.7)	2.00 (1.25,3.19)	0.004	1.85 (1.07,3.22)	0.029
*P* for trend				0.003		0.020
**Categories**						
Q1 (<254.2)	146	50 (34.2)	1.00 (1.00,1.00)	Ref	1.00 (1.00,1.00)	Ref
Q2–Q4 (≥254.2)	436	241 (55.3)	2.24 (1.52,3.30)	<0.001	2.22 (1.41,3.50)	<0.001

1 *ORs of all-cause mortality in relation to serum concentrations of serine quartiles were calculated with the use of conditional logistic regression models. Q, quartile; Ref, reference.*

2 *Adjusted for age, sex, body mass index, treatment group, MTHFR C677T, smoking status, alcohol drinking status, systolic blood pressure, diastolic blood pressure, fasting blood glucose, total cholesterol, triglycerides, HDL-C, eGFR, total homocysteine at baseline, vitamin B12, serum folate at baseline, and time-averaged BP, during the treatment period*.

### Stratification Analysis

Table 3 shows the results of the stratification analyses in various subgroups with serine modeled as a quartile variable, and compared Q1 to combined Q2–Q4(<254.2 vs. ≥254.2 μ mol/L). As shown in the table, after adjusting for the related variables, the majority of the variables including age, sex, BMI, smoking and drinking status, blood pressure, treatment group, MTHFR C677T genotype, blood lipids, HDL, diabetes status, baseline serum folate and baseline vitamin B12 levels have no modifiable effects on the relationship between serine and mortality.

**Table 3:** The association between serum serine and the risk of all-cause mortality in various subgroups.

**Subgroups**	**Serine, μ mol/L**				**OR(95%CI)**	***P*** **for interaction**
	**Q1 (<254.2)**		**Q2–Q4 (≥254.2)**			
	***N***	**Cases (%)**	***N***	**Cases (%)**		
Age, y						0.199
<64.5 (median)	69	28 (40.6)	222	117 (52.7)	1.83 (0.98, 3.41)	
≥64.5	77	22 (28.6)	214	124 (57.9)	3.12 (1.67, 5.81)	
Sex						0.509
Female	58	21 (36.2)	196	106 (54.1)	2.17 (1.10, 4.28)	
Male	88	29 (33.0)	240	135 (56.2)	2.82 (1.57, 5.07)	
BMI, kg/m^2^						0.141
<24.5 (median)	71	29 (40.8)	220	126 (57.3)	1.97 (1.06, 3.66)	
≥24.5	75	21 (28.0)	216	115 (53.2)	3.96 (2.04, 7.67)	
Smoking						0.777
Never	75	26 (34.7)	241	132 (54.8)	2.36 (1.31, 4.27)	
Former	26	8 (30.8)	44	26 (59.1)	5.54 (1.20, 25.50)	
Current	45	16 (35.6)	151	83 (55.0)	3.08 (1.27, 7.48)	
Alcohol drinking						0.848
Never	82	30 (36.6)	250	139 (55.6)	2.54 (1.42, 4.53)	
Former	15	5 (33.3)	50	30 (60.0)	3.51 (0.39, 31.57)	
Current	49	15 (30.6)	136	72 (52.9)	3.15 (1.36, 7.30)	
Systolic BP at baseline, mmHg						0.650
<168.0 (median)	64	20 (31.2)	223	111 (49.8)	3.19 (1.59, 6.37)	
≥ 168.0	82	30 (36.6)	213	130 (61.0)	2.57 (1.42, 4.65)	
Diastolic BP at baseline, mmHg						0.684
<94.0 (median)	71	24 (33.8)	215	111 (51.6)	2.37 (1.24, 4.54)	
≥94.0	75	26 (34.7)	221	130 (58.8)	2.53 (1.40, 4.58)	
Treatment group						0.286
Enalapril	84	25 (29.8)	226	130 (57.5)	3.30 (1.78, 6.10)	
Enalapril-folic acid	62	25 (40.3)	210	111 (52.9)	2.09 (1.08, 4.04)	
MTHFR C677T						0.182
CC	28	6 (21.4)	113	62 (54.9)	4.06 (1.32, 12.54)	
CT	71	22 (31.0)	221	122 (55.2)	2.99 (1.60, 5.57)	
TT	47	22 (46.8)	102	57 (55.9)	1.20 (0.51, 2.83)	
eGFR, mL/(min·1.73 m^2^)						0.018
<91.2 (median)	73	19 (26.0)	211	121 (57.3)	4.42 (2.25, 8.69)	
≥91.2	70	30 (42.9)	215	112 (52.1)	1.32 (0.72, 2.44)	
Total cholesterol, mmol/L						0.570
<5.6 (median)	76	25 (32.9)	208	118 (56.7)	2.81 (1.53, 5.17)	
≥5.6	67	24 (35.8)	218	115 (52.8)	2.27 (1.18, 4.37)	
Triglycerides, mmol/L						0.084
<1.4 (median)	65	27 (41.5)	223	121 (54.3)	1.80 (0.97, 3.36)	
≥ 1.4	80	23 (28.7)	209	117 (56.0)	3.35 (1.81, 6.22)	
HDL cholesterol, mmol/L						0.807
<1.3 (median)	75	27 (36.0)	208	115 (55.3)	2.38 (1.29, 4.38)	
≥ 1.3	70	23 (32.9)	225	124 (55.1)	2.39 (1.29, 4.45)	
Diabetes						0.878
Non-DM	121	41 (33.9)	358	191 (53.4)	2.46 (1.54, 3.93)	
DM	23	8 (34.8)	69	43 (62.3)	1.67 (0.36, 7.83)	
tHcy, μmol/L						0.012
<13.7 (median)	65	27 (41.5)	220	110 (50.0)	1.38 (0.74, 2.58)	
≥ 13.7	78	22 (28.2)	208	125 (60.1)	4.76 (2.43, 9.32)	
Folate, ng/mL						0.056
<6.5 (median)	63	19 (30.2)	220	125 (56.8)	3.58 (1.84, 6.95)	
≥6.5	78	29 (37.2)	206	107 (51.9)	1.48 (0.82, 2.68)	
Vitamin B12, pg/mL						0.753
< 374.7 (median)	78	27 (34.6)	205	112 (54.6)	2.55 (1.39, 4.67)	
≥374.7	63	21 (33.3)	221	120 (54.3)	3.08 (1.56, 6.08)	
5-MTHF, ng/mL						0.048
<6.1 (median)	68	23 (33.8)	219	135 (61.6)	4.10 (2.09, 8.05)	
≥6.1	74	26 (35.1)	214	105 (49.1)	1.83 (0.98, 3.40)	

*Adjusted, if not stratified, for age, sex, body mass index, treatment group, MTHFR C677T genotype, smoking status, alcohol drinking status, systolic blood pressure, diastolic blood pressure, fasting blood glucose, total cholesterol, triglycerides, HDL-C, eGFR, total homocysteine at baseline, total folate at baseline, vitamin B12, and time-averaged BP during the treatment period. DM, diabetes mellitus*.

We observed interactions between serum serine and baseline tHcy levels [<13.7 (median) vs. ≥13.7 μ mol/L] (*P* for interaction=0.012), baseline 5-MTHF levels [<6.1 (median) vs. ≥6.1 ng/mL] (*P* for interaction=0.048), as well as eGFR levels [<91.2 (median) vs. ≥91.2 mL/(min per 1.73 m^2^)] (*P* for interaction =0.018) on all-cause mortality (Table 3). Higher serum serine levels were significantly associated with a higher risk of all-cause mortality in those with eGFR <91.2 mL/(min per 1.73 m^2^) (OR, 4.42; 95% CI, 2.25–8.69), tHcy ≥13.7 μ mol/L (OR, 4.76; 95% CI, 2.43–9.32), and 5-MTHF <6.1 ng/mL (OR, 4.10; 95% CI, 2.09–8.05).

## Discussion

In this cohort of hypertensive Chinese adults, we found that the risk of all-cause mortality first increased sharply, and then tended to flatten. After adjusting for possible related confounders, the risk of mortality still increased significantly with the increase in serine levels. Compared with group Q1, the mortality risks of groups Q2, Q3 and Q4 were significantly increased [ORs, 95% CI: Q2: 2.32, (1.32–4.07); Q3: 2.59, (1.48–4.54); and Q4: 1.85, (1.07–3.22)]. To the best of our knowledge, this study is the first to illustrate the potential correlations between serum serine levels and all-cause mortality in a longitudinal cohort.

There are few studies concerning serum serine levels predicting the risk of mortality. Teymoori et al. evaluated the association between dietary serine intakes and hypertension incidents, and observed that 10% of the cohort subjects (429) incident cases of hypertension were ascertained after 3 years of follow-up. The OR of the highest quartile of serine intake was 1.43 (95% CI: 1.05–1.95; *P* for trend: 0.03) compared to the lowest adjusted for age and sex (14). Studies have reported that majorly depressed (15) and schizophrenic patients have significantly increased plasma serine levels compared to normal control groups (16), these psychiatric disorders may contribute to all-cause mortality in the long term. Gu et al. reported that in the serum samples, serine levels and their metabolites were elevated in the colorectal cancer group compared to that of the control group (17). Cadoni et al. found higher serine was significantly associated with decreased overall survival and increased risk of the advanced stage in head and neck cancer (8).

However, the previous findings are inconsistent. Kinny-Köster et al. demonstrated that plasma branched-chain and aromatic amino acids are promising additional biomarkers to determine the increased risk of mortality in patients with end-stage liver disease, but serine showed no significant correlations in survival analysis (11), besides, Mustafa et al. investigated the serum amino acids profiles, demonstrated that serum serine levels were significantly decreased in patients with renal cell carcinoma (RCC) compared to the age and sex matched controls (18), they assumed that the underlying reason would be that kidney tumors might be affecting the reabsorption of amino acids by affecting overall renal function, and the declined serine levels is a consequent result of the RCC.

The clinical characteristics of the study population may account for the aforementioned inconsistent epidemiological research observations, therefore, it is necessary to further examine whether reported associations exist stably in different race and ethnic groups.

Serine can be derived from four possible sources: dietary intake; biosynthesis from the glycolytic intermediate 3-phosphoglycerate; from glycine; and by protein and phospholipid degradation (19), a study showed that subjects belonging to different habitual diet groups have significantly different plasma concentrations of many amino acids, but the plasma serine concentrations were less marked (20). Actually, the *de novo* synthesis of serine is critical, as dietary serine contributes little or nothing to serine metabolism (19), thus it is insufficient to meet the demands of whole body serine homeostasis (21). We speculated that the disturbance of serine homeostasis may attribute to its aberrant biosynthesis pathway, rather than dietary habit or food components of people, this may the different characteristic of serine, a non-essential amino acid, distinguishes from essential amino acids.

A possible biological explanation for our finding would be the roles of serine in the pathological process. Serine is a critically important “input” of one-carbon metabolism and nucleotide biosynthesis, as a hub of one-carbon metabolism and therefore, its overexpression is an important feature of different malignancies (22, 23). The enzyme of serine biosynthesis, phosphoglycerate dehydrogenase (PHGDH) is overexpressed in various types of cancer (24). It seems that cancer cells have a high demand for serine, the flux toward serine synthesis is up-regulated in breast cancer has been observed (25, 26). A study demonstrated that the flux of serine synthesis from 3-phosphoglycerate exhibits a positive correlation with the proliferation rate of tumor-derived cell lines (27). Additionally, it was proved the synthesis and transport pathways genes of serine were overexpressed or up-regulated in various types of cancer (24–26, 28). Combined with the chronic disease, hypertension, aberrant metabolism in the pathological conditions accompanied by the increased serum serine levels may be the reasons promote to the development of diseases and, in the long term, the increased mortality risk.

Some interesting findings of particular note are the potential effect modifiers (Table 3): baseline tHcy and 5-MTHF, a plausible biological explanation for the interaction from tHcy and 5-MTHF is that higher tHcy, which is usually resulted from lower circulating folate and 5-MTHF, is associated with many dangerous and lethal diseases, such as cardiovascular disease (29), stroke (30), and Alzheimer's disease (31). Besides, we found that eGFR negatively modified the effect of serine on the all-cause mortality, previous research showed a significantly greater risk for both all-cause and cardiovascular mortality in the lower eGFR group compared with the stable group over a median of 7.2 years after the last eGFR measure (32) in elderly treated hypertensive patients. If these results are further confirmed, maintaining low tHcy, high 5-MTHF, high eGFR, and low serine levels could be a highly effective strategy for reducing the risk of mortality.

One advantage of this study is we were able to obtain an accurate measurement of the serum serine, as compared with the fortified amino acid dietary intervention method utilized in a study by Verhoef et al. (33), the serum serine levels measured in our study are more directly and precisely reflect the actual available amount for physiological activities or molecular mechanisms, while the amount of serine obtained from dietary intake may be influenced by the food matrix, the bio-accessibility and bioavailability of the amino acid. Meanwhile, several limitations should be noted. First, the existence of the enzyme DL-serine racemase (EC 5.1.1.10) has been reported to directly convert L-serine into D-serine (34), two enantiomers may possess different effects on the health and disease status (35), however the serine levels we determined in this study only present the total serine. Second, our current study was conducted in a Chinese hypertensive population, thus whether the observed findings can be extrapolated or applicable to other populations needs further investigation. Third, due to a lack of data on specific causes of death, the present study could not further explore cause-specific mortality. In addition, despite the comprehensive adjustment for confounders, we cannot exclude the possibility of residual confounding by related dietary factors or other variables. This study provides a key piece of evidence toward the importance of evaluating serum serine levels when assessing the risk of mortality.

## Conclusion

In summary, our study observed for the first time that the levels of baseline serum serine are a risk factor in increasing all-cause mortality. To explore these correlations in greater depth, further experimental studies and clinical trials are required. Our results suggest that serine levels should be considered as a potential marker for screening risk factors of mortality, in both clinical practice and public health settings, but this finding needs to be validated and confirmed in future investigations with larger populations.

## Data Availability Statement

The original contributions presented in the study are included in the article/supplementary materials, further inquiries can be directed to the corresponding author/s'.

## Ethics Statement

The studies involving human participants were reviewed and approved by the Ethics Committee of the Institute of Biomedicine, Anhui Medical University, Hefei, China (FWA assurance number FWA00001263). The patients/participants provided their written informed consent to participate in this study.

## Author Contributions

XX, XQ, QH, and PQ conceived and designed the experiments. XX, YD, BW, XQ, and YS conducted the study. PC performed the quantification of serum samples. PQ, QL, NZ, ZZ, and YW collected and analyzed the data. QH, NZ, ZW, and ZZ drafted the manuscript. All authors contributed to the article and approved the submitted version.

## Funding

This work was supported by National Key Research and Development Program grants 2016YFE0205400 (to XX), 2018ZX09739010 (to XX), and 2018ZX09301034003 (to XX); Science and Technology Planning Project of Guangzhou, China grant 201707020010 (to XX); Science, Technology and Innovation Committee of Shenzhen grants GJHS20170314114526143 (to XX) and JSGG20180703155802047 (to XX); and Economic, Trade and Information Commission of Shenzhen Municipality grants 20170505161556110 (to XX), 20170505160926390 (to XX), and 201705051617070 (to XX).

## Conflict of Interest

YS was employed by Shenzhen AUSA Pharmed Co Ltd. The remaining authors declare that the research was conducted in the absence of any commercial or financial relationships that could be construed as a potential conflict of interest.

## Publisher's Note

All claims expressed in this article are solely those of the authors and do not necessarily represent those of their affiliated organizations, or those of the publisher, the editors and the reviewers. Any product that may be evaluated in this article, or claim that may be made by its manufacturer, is not guaranteed or endorsed by the publisher.

## Abbreviations

eGFR: estimated glomerular filtration rate
tHcy: total homocysteine
NCC: nested case-control
SAM: S-adenosyl methionine
CSPPT: the China Stroke Primary Prevention Trial
SD: standard deviation
OR: odds ratio
CI: confidence interval
BMI: body mass index
SBP: systolic blood pressure
DBP: diastolic blood pressure
MTHFR: methylenetetrahydrofolate reductase
HDL-C: high density lipoprotein cholesterol
DM: diabetes mellitus.
